# Comparative analysis of chromosomal localization of ribosomal and telomeric DNA markers in three species of Pyrgomorphidae grasshoppers

**DOI:** 10.3897/CompCytogen.v11i4.14066

**Published:** 2017-09-12

**Authors:** Olesya G. Buleu, Ilyas Y. Jetybayev, Alexander G. Bugrov

**Affiliations:** 1 Novosibirsk State University, Pirogova Str. 2, Novosibirsk 630090 Russia; 2 Institute of Systematics and Ecology of Animals, Russian Academy of Sciences, Siberian Branch, Frunze str. 11, 630091 Novosibirsk, Russia; 3 Institute of Cytology and Genetics, Russian Academy of Sciences, Siberian Branch, Pr. Lavrentjeva 10, 630090 Novosibirsk, Russia

**Keywords:** Pyrgomorphidae grasshoppers, karyotype, C-banding, FISH, 28S rDNA, telomeric DNA

## Abstract

The karyotypes of three species of Pyrgomorphidae grasshoppers were studied: *Zonocerus elegans* (Thunberg, 1815), *Pyrgomorpha guentheri* (Burr, 1899) and *Atractomorpha lata* (Mochulsky, 1866). Data on karyotypes of *P. guentheri* and *Z. elegans* are reported here for the first time. All species have karyotypes consisting of 19 acrocentric chromosomes in males and 20 acrocentric chromosomes in females (2n♂=19, NF=19; 2n♀=20, NF=20) and X0/XX sex determination system. A comparative analysis of the localization of C-heterochromatin, clusters of ribosomal DNA, and telomere repeats revealed inter-species diversity in these cytogenetic markers. These differences indicate that the karyotype divergence in the species studied is not associated with structural chromosome rearrangements, but with the evolution of repeated DNA sequences.

## Introduction

Orthoptera is undoubtedly one of the most well cytogenetically studied groups of insects. Even at an early stage of comparative cytogenetics, they became convenient research models for analysis of mitotic and meiotic chromosomes. It was through working on Orthoptera that Robertson (1916) established the main tendencies in insect karyotype evolution through centric fusion of chromosomes, Darlington (1931, 1932) described meiosis in detail, and White (1968, 1973) proposed the chromosome speciation hypothesis.

However, the karyotypic features of various Orthoptera groups have been studied extremely unevenly. Among Acridoidea and Pyrgomorphoidea, only the family Acrididae can be considered as well studied, whereas the karyotypes of Pyrgomorphidae, Pamphagidae, Lathiceridae, Lentulidae and some other families remain poorly investigated or not studied at all. Analysis of chromosome sets within such Orthoptera groups, which have never been studied before, in conjunction with the use of new techniques for chromosome research, may therefore potentially lead to many new insights. As an example, using molecular cytogenetic methods, in-depth research of the family Pamphagidae has recently revealed new evolutionary pathways of autosomes and sex chromosomes previously unknown in this family (Bugrov et al. 2016, Jetybayev et al. 2017).

The basal chromosome set of the family Pyrgomorphidae (superfamily Pyrgomorphoidea) coincides with that of the family Pamphagidae (superfamily Acridoidea) and contains 19 acrocentric chromosomes in males, 20 in females (sex determination X0/XX) (White 1973, Hewitt 1979). In this regard, these two families with FN=19♂/20♂ differ from other Acridoidea species, the basal karyotype of which contains 23 acrocentric chromosomes in males, 24 in females (sex determination X0/XX FN=23♂/24♀). The morphological similarity of the modal chromosome set in Pamphagidae and Pyrgomorphidae was noted a long time ago (White 1973, Hewitt 1979); however, the question as to whether this similarity represents a phylogenetic signal is still unknown. This is partially related to the poor degree of karyological study of Pyrgomorphidae grasshoppers. The karyotypes of only about 30 species are known from tropical and subtropical regions of the Old World (Makino 1951, Sannomiya 1973; Nankivell 1976, John and King 1983, Fossey et al. 1989, Williams and Ogunbiyi 1995, Seino et al. 2013, Seino and Dongmo 2015). The vast majority of species have a 19-chromosome karyotype, but a few species have been shown to have a different karyotype, resulting from one, two or three Robertsonian translocations (White 1973, Fossey et al. 1989). Moreover, only in a few species the C-heterochromatin localization has been studied (*Atractomorpha similis*, *A. hypoestes*, *A. austraIis*; *Pyrgomorpha conica*) (Nankivell 1976, John and King 1983, Suja et al. 1993).

Molecular cytogenetic studies were previously performed for only one species of Pyrgomorphidae – *Pyrgomorpha conica* (Suja et al. 1993, López-Fernández et al. 2004, 2006).

The aim of the present study, therefore, is to reveal new features of chromosome sets in as-yet unstudied species of Pyromorphidae grasshoppers. We used standard cytogenetic techniques, as well as molecular-cytogenetic methods, to find additional markers of linear chromosome differentiation. The fluorescence *in situ* hybridization (FISH) method was employed to localize functionally important regions in autosomes and the sex chromosomes, containing clusters of ribosomal DNA and telomeric (TTAGG)*_n_* repeats. The choice of these molecular markers was prompted by an awareness of their important functional role in the genome and chromosome localization of many insects including Pyrgomorphidae grasshoppers (Sahara et al. 1999, López-Fernández et al. 2004, Cabrero and Camacho 2008), and renders the data reported herein suitable for comparative analysis.

## Material and methods

### Material collection, fixation and C-banding

Three species belonging to Pyrgomorphidae were studied: 1) *Zonocerus elegans* (Thunberg, 1815) (Phymateini tribe) – six males of this species collected during February and March 2003 in South Africa, in vicinity of Springbok city; 2) *Pyrgomorpha guentheri* (Burr, 1899), (Pyrgomorphini tribe) – five males of this species collected in June 2007 in Armenia; 3) *Atractomorpha lata* (Mochulsky, 1866) (Atractomorphini tribe) – two males of this species collected in August, 2005 on Ishigaki island (Ryukyu Archipelago, Japan).

The collected insects were injected with 0.1% colchicine solution and, after 1.5–2.0 hours, their testes were dissected and placed into 0.9% solution of sodium citrate for 20 minutes, then fixed in 3:1 ethanol:glacial acetic acid for 15 minutes. The fixed material was rinsed and kept in 70% ethanol.

C-banding of the chromosome preparations was performed according to the protocol of Sumner (1972), with minor modifications.

### Fluorescence *in situ* hybridization (FISH)

Fluorescence *in situ* hybridization on meiotic chromosomes was carried out according to the protocol of Pinkel (1986) with modifications (Rubtsov et al. 2000, 2002). The rDNA probe was obtained as was described earlier (Jetybayev et al. 2017). The sequences of primers used for 28S rDNA were designed on the basis of consensus sequence of the 28S rRNA gene, obtained by the alignment of rDNA sequences of different species of grasshoppers (gb|AY859546.1, gb| KM853499.1, gb|AY125286.1 and gb|EU414723.1), using the software packages PerlPremier (Marshall 2004) and Mulalin (Corpet 1988) (Table 1). The DNA probe for detection of telomeric repeats (TTAGG)*_n_* in metaphase chromosomes was generated with non-template PCR (Ijdo et al. 1991) with 5'-TAACCTAACCTAACCTAACC-3' and 5'-TTAGGTTAGGTTAGGTTAGG-3' primers according to standard protocol (Sahara et al. 1999). DNA labelling was performed in additional PCR cycles with Tamra-5-dUTP and Fluorescein-12-dUTP (Biosan, Novosibirsk, Russia).

**Table 1.** Primers used for 28S rDNA amplification.

Name	Sequence	Amplicon size
28SrDNA1F	5’-TGGACAATTTCACGACCCGTC-3’	600 bp
28SrDNA1R	5’-GCGTTTGGTTCATCCCACAG-3’	
28SrDNA2F	5’-TGAACCAAACGCCGAGTTAAGG-3’	650 bp
28SrDNA2R	5’-ATTCCAGGGAACTCGAACGCTC-3’	
28SrDNA3F	5’-TTCTGCATGAGCGTTCGAGTTC-3’	700 bp
28SrDNA3R	5’-TGGGCAGAAATCACATTGCGTC-3’	

Chromosome counterstaining was preformed after FISH with 4´,6-diamidino-2-phenylindole (DAPI) using Vectashield antifade containing 200 ng/ml DAPI.

### Microscope analysis

Microscopic analysis was performed at the Center for Microscopy of Biological Objects (Institute of Cytology and Genetics, Novosibirsk, Russia). Chromosomes were studied with an AxioImager M1 (Zeiss) fluorescence microscope equipped with filter sets #49, #46HE, #43HE (Zeiss) and a ProgRes MF (MetaSystems) CCD camera. The ISIS5 software package (MetaSystems GmbH, Germany) was used for image capture and analysis.

### Chromosome nomenclature

The nomenclature suggested for grasshoppers (King and John 1980, Santos et al. 1983, Cabrero et al. 1985) was used in the description of chromosomes, karyotypes and C-banding.

## Results

### Karyotype

Data on karyotypes of *P. guentheri* and *Z. elegans* are reported for the first time. Karyotype of *A. lata* was described earlier (Makino 1951). The karyotype reference for this species, reported from Cameroon (Seino et al. 2014), requires verification, given that the distribution of this species is restricted to South-East Asia (http://orthoptera.speciesfile.org).

Diploid sets (2n) of chromosomes in all species studied consisted of 19 (♂) and 20 (♀) acrocentric chromosomes. Sex determination was X0 male and XX female. The karyotype structure consists of three large (L_1_–L_3_), five medium (M_4_–M_8_) and one small (S_9_) pair of autosomes. The fundamental number of chromosome arms (FN) was 19 in male and 20 in female.

**Figure 1.**
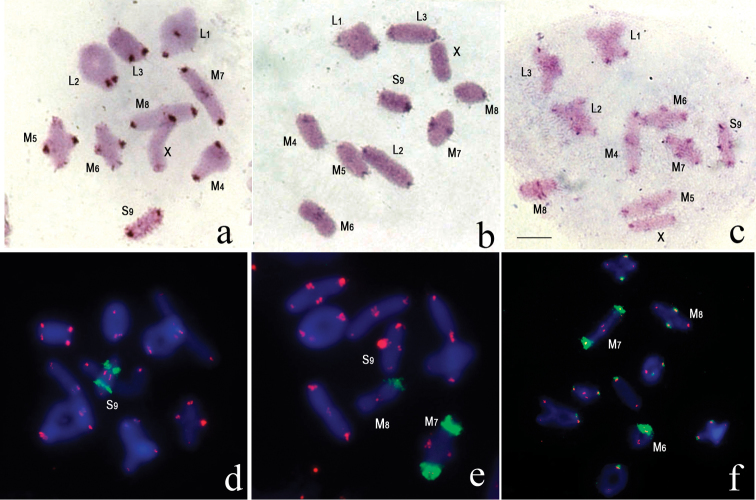
C-banding (**a–c**) and fluorescence *in situ* hybridization of 28S ribosomal DNA (green) and telomere (TTAGG)*_n_* (red) probes (**d–f**) with chromosomes of: **a, d** *Zonocerus elegans*, metaphase I of meiosis **b, e** *Atractomorpha lata*, metaphase I of meiosis **c, f** *Pyrgomorpha guentheri*, metaphase I of meiosis Bar = 5 µm.

The large autosomes of *Z. elegans* and *A. lata* were distinctly different from each other, while the large chromosome pairs (L_1_–L_3_) of *P. guentheri* and *A. lata* were almost equal in size (Fig. 1a, b, c). The medium and small autosomes varied slightly in size and represented a gradually decreasing size range. All the species studied had a large acrocentric X chromosome, which was almost equal to the L_1_ chromosome (Fig. 1a, b, c). At meiotic prophase in *Z. elegans* and *A. lata*, each large bivalent usually formed two, rarely one chiasmata, while medium and small bivalents formed one chiasma (Fig. 1a, b, d, e). In *P. guentheri* each bivalent formed only one chiasma (Fig. 1c, f).

### C-banding

In the karyotype of *Zonocerus elegans*, C-banding revealed large paracentromeric C-blocks in all chromosomes of the set. Small terminal C-positive blocks were localized in M_5_, M_6_, M_7_ medium size autosome pairs and the X chromosome. The S_9_ autosome is megameric: multiple small C-heterochromatin blocks are located within the whole autosome length (Fig. 1a).

In *Atractomorpha lata*, medium sized pericentric C-blocks were revealed in the L_1_–L_3_, M_7_ and S_9_ autosome pairs. The rest of the medium sized autosomes (M_4_, M_5_, M_6_, M_8_) and X chromosome had small pericentric C-blocks. In L_1_, M_4_, M_8_ and S_9_ bivalents the pericentromeric C-blocks exhibited variation in size in homologous chromosomes. On one of the chromosomes in these bivalents pericentromeric C-block was large, while on the other chromosome it was small (Fig. 1b).

C-banding of *Pyrgomorpha guentheri* chromosomes revealed a medium sized pericentromeric C-block in all autosomes with the exception of the L_3_ pair, which had a small block. The pericentromeric C-block on the X chromosome was small. Medium sized terminal C-blocks were found in M_4,_ M_6_, M_7_, M_8_, S_9_ chromosomes (Fig. 1c).

### Fluorescence *in situ* hybridization (FISH) of chromosomes with ribosomal and telomeric DNA probes

Analysis of fluorescence *in situ* hybridization of telomeric DNA-probes showed that in all the species studied, telomeric repeats were localized only in terminal areas of all chromosomes. In *Atractomorpha lata* FISH revealed difference in the size of telomeric cluster in a small pair (S_9_). Fluorescent signal was significantly stronger on one of the homologous chromosomes in S_9_ bivalent (Fig. 1e).

FISH of the 28S ribosomal DNA probe revealed interspecific variation of rDNA localization. In *Zonocerus variegatus*, clusters of rDNA were localized in the interstitial region of the S_9_ autosome (Fig. 1d). In *Atractomorpha lata*, rDNA clusters were observed in pericentromeric regions of two pairs of autosomes (M_7_, M_8_). In one specimen, in M_8_ pair the rDNA cluster was observed only on one of the homologous chromosomes in the bivalent (Fig. 1e). In *Pyrgomorpha guentheri* rDNA clusters were localized in the pericentromeric region of all chromosomes. Most of the rDNA clusters were small, whereas the clusters in the M_6,_ M_7,_ and M_8_ chromosome pairs were large (Fig. 1f).

## Discussion

Comparative analysis of karyotypes of three species of Pyrgomorphidae grasshoppers from the Ethiopian, Mediterranean and East Asian regions confirms that 2n♂=19 (NF=19), 2n♀=20 (NF=20) (X0/XX sex determination) is the basal chromosome set in this group. However, differences from the basal chromosomal set were also observed. Some species exhibit one (*Sphenarium mexicanum*, 2n♂=17), three (*Pyrgomorpha granulata*, 2n♂=13) or four (*Pyrgomorpha rugosa*, 2n♂=11) Robertsonian translocations (White 1973, Fossey et al. 1989). Another variant of non-basal karyotype was described in *Pyrgomorpha* sp. White (1973), referring to his unpublished data, mentions that the 2n=18, XX♀/XY♂ karyotype in this species resulted from centric fusion of acrocentric X-chromosome and acrocentric autosome. In all the cases mentioned above, the fundamental karyotype number remains constant: NF♂=19, NF♀=20.

Searching for new karyotype variants in this poorly studied group holds the potential to turn up interesting findings. For instance, recently, a new model of the Y-chromosome evolution was proposed based on studies in Pamphagidae grasshoppers. It was shown that in Pamphagidae grasshoppers centric fusion of the X chromosome and autosome occurred independently in two phylogenetic branches, and due to further evolution the neo-Y chromosome exhibited different stages of degradation process (Bugrov et al. 2016, Jetybayev et al. 2017).

The Pyrgomorphidae and Pamphagidae both have NF♂=19, NF♀=20, while Acridoidea has NF♂=23, NF♀=24. This gives rise to a question about the monophyly or homoplasy of Pyrgomorphidae and Pamphagidae. However, further detailed analysis of linear chromosome differentiation in these families is needed to shed light on this issue.

The present study revealed the difference in size and localization of C-positive blocks of chromosomes between the species studied. Furthermore, in *A. lata* and *P. guentheri* the difference observed on homologous chromosomes suggests the presence of the polymorphism in population of these species. A high level of interpopulation polymorphism of C-positive regions was previously reported for three Pyrgomorphidae species from Australia, Papua-New Guinea and Indonesia (Nankivell 1976, John and King 1983). Different populations of *Atractomorpha crenaticeps*, *A. similis* and *A. australis* were found to show polymorphism in terms of the size and localization of C-blocks in pericentromeric, interstitial, and telomeric regions in large and medium chromosomes. Furthermore, in some populations of *A. australis* additional arms were found, consisting of very large C-heterochromatin. Later some supernumerary heterochromatic segments in two chromosome pairs were revealed in *Pyrgomorpha conica* (Suja et al. 1993).

Such diversity in terms of the size and localization of C-positive blocks within different species of Pyrgomorphidae grasshoppers indicates that the evolution of repeated DNA sequences plays an important role in the divergence of karyotypes in this group.

However, molecular cytogenetic studies of repetitive sequences in chromosomes of Pyrgomorphidae grasshoppers were carried out only in *Pyrgomorpha conica* (Suja et al. 1993, López-Fernández et al. 2004, 2006). These methods showed that supernumerary heterochromatic segments derived from amplification of rRNA genes (Suja et al. 1993) and telomeric repeats enrich pericentric C-positive blocks (López-Fernández et al. 2006).

The current study represents comparative analysis of localization of 28S rDNA and telomeric (TTAGG)*_n_* sequences in this group. Telomeric repeats exhibited very conservative localization, only in terminal areas of all chromosomes, and no interstitial telomeric sites. This may indicate that the karyotype evolution of these species did not include chromosome structural reorganizations involving terminal regions of chromosomes (for example pericentric inversions). However, interstitial telomeric sequences have previously been reported for Acrididae grasshoppers; such localization of clusters of telomeric DNA may be the result of such chromosomal reorganizations (Jetybayev et al. 2012). The observed polymorphism in the size of the telomeric cluster in *A. lata* correlates with C-block polymorphism in S_9_ chromosome. Previously the same kind of polymorphism in terms of size was reported for *Pyrgomorpha conica* (López-Fernández et al. 2006). The C-blocks consist of amplified repetitive sequences, and sometimes amplification could involve telomeric or rDNA repeats.

Fluorescence hybridization *in situ* (FISH) of the rDNA fragment revealed a consistent pattern of rDNA distribution in chromosomes of the Pyrgomorphidae family. Ribosomal DNA clusters may be found in one pair (S_9_ in *Z. elegans*), two pairs (M_7_, S_9_, in *A. lata*) or in all chromosomes (the pericentric regions of chromosomes in *P. guentheri*). However, in *P. guentheri* most of the rDNA clusters were very small and only clusters on the chromosomes M_7_, M_8_ and S_9_ were significantly larger. This might be the result of a recent expansion of rDNA repeats in pericentric heterochromatin and the newly arisen rDNA clusters may be silent (Suja et al. 1993, Cabrero and Camacho 2008, Jetybayev et al. 2012).

The diversity in the rDNA distribution itself apparently reflects the degree of divergence in the species studied, which belong to different tribes of Pyrgomorphidae. Comparing the patterns of rDNA distribution in the karyotypes of the species studied here with known data on rDNA distribution in karyotypes of other Orthoptera, we may suggest that Pyrgomorphidae are close to the Acrididae family of Orthoptera. In this family, distribution of rDNA is basically limited to one or two pairs of chromosomes in the karyotype (Cabrero and Camacho 2008). In single cases, clusters of rDNA were revealed in the pericentric heterochromatin of all chromosomes in the set (Jetybayev et al. 2012). In contrast to Pyrgomorphidae and Acrididae grasshoppers, multiple localization of rDNA clusters on one chromosome in the Pamphagidae family has been shown (Bugrov et al. 2016, Jetybayev et al. 2017). Perhaps, the revealed differences in the localization of rDNA in Pyrgomorphidae and Acrididae on the one hand, and Pamphagidae on the other hand, may contain a certain phylogenetic signal. However, we still lack enough data, especially for the Pyrgomorphidae family, to approach the problem of the origin of the modal 19-chromosome karyotype of Pyrgomorphidae and Pamphagidae from a molecular-cytogenetic position. Nevertheless, intensive development of molecular-cytogenetic methods gives us hope that more species examined will allow further reconsideration of the pathways of Orthoptera chromosome evolution, which led to the formation of similar karyotype structure of Pyrgomorphidae and Pamphagidae grasshoppers.
